# Reproducibility of compartmental modelling of ^18^F-FDG PET/CT to evaluate lung inflammation

**DOI:** 10.1186/s40658-019-0265-8

**Published:** 2019-12-16

**Authors:** Laurence D. Vass, Sarah Lee, Frederick J. Wilson, Marie Fisk, Joseph Cheriyan, Ian Wilkinson

**Affiliations:** 1 Experimental Medicine and Immunotherapeutics, Department of Medicine, Addenbrookes Hospital, Cambridge, UK; 2 Amallis Consulting LTD, London, UK; 3 GSK R &D, Brentford, UK; 4 Cambridge University Hospitals NHS Trust, Cambridge, UK

**Keywords:** Positron emission tomography computed tomography, Kinetic modelling, Lung inflammation, Fluorodeoxyglucose F18, Reproducibility of results, Pulmonary disease, Chronic obstructive, Biomarkers

## Abstract

**Introduction:**

Compartmental modelling is an established method of quantifying ^18^F-FDG uptake; however, only recently has it been applied to evaluate pulmonary inflammation. Implementation of compartmental models remains challenging in the lung, partly due to the low signal-to-noise ratio compared to other organs and the lack of standardisation. Good reproducibility is a key requirement of an imaging biomarker which has yet to be demonstrated in pulmonary compartmental models of ^18^F-FDG; in this paper, we address this unmet need.

**Methods:**

Retrospective subject data were obtained from the EVOLVE observational study: Ten COPD patients (age =66±9; 8M/2F), 10 *α*_1_ATD patients (age =63±8; 7M/3F) and 10 healthy volunteers (age =68±8; 9M/1F) never smokers. PET and CT images were co-registered, and whole lung regions were extracted from CT using an automated algorithm; the descending aorta was defined using a manually drawn region. Subsequent stages of the compartmental analysis were performed by two independent operators using (i) a MIAKAT^TM^ based pipeline and (ii) an in-house developed pipeline. We evaluated the metabolic rate constant of ^18^F-FDG (*K*_*im*_) and the fractional blood volume (*V*_*b*_); Bland-Altman plots were used to compare the results. Further, we adjusted the in-house pipeline to identify the salient features in the analysis which may help improve the standardisation of this technique in the lung.

**Results:**

The initial agreement on a subject level was poor: Bland-Altman coefficients of reproducibility for *K*_*im*_ and *V*_*b*_ were 0.0031 and 0.047 respectively. However, the effect size between the groups (i.e. COPD, *α*_1_ATD and healthy subjects) was similar using either pipeline. We identified the key drivers of this difference using an incremental approach: ROI methodology, modelling of the IDIF and time delay estimation. Adjustment of these factors led to improved Bland-Altman coefficients of reproducibility of 0.0015 and 0.027 for *K*_*im*_ and *V*_*b*_ respectively.

**Conclusions:**

Despite similar methodology, differences in implementation can lead to disparate results in the outcome parameters. When reporting the outcomes of lung compartmental modelling, we recommend the inclusion of the details of ROI methodology, input function fitting and time delay estimation to improve reproducibility.

## Introduction

Inflammation is a hallmark of many respiratory diseases and is thought to be complicit in the progression of chronic lung diseases such as chronic obstructive pulmonary disease (COPD). The limitations of clinical measurements to quantify pulmonary inflammation are well documented [1]. ^18^F-FDG PET-CT is a non-invasive imaging technique; quantification of ^18^F-FDG uptake has emerged as a promising biomarker to assess lung inflammation [2]. Recruitment of inflammatory cells requires increased glucose utilisation; therefore, ^18^F-FDG uptake should be elevated in inflammatory pathologies [3].

A major challenge in quantifying ^18^F-FDG in the lung is the poor signal-to-noise ratio; the low basal uptake of FDG is a consequence of the low density of lung tissue (due to large proportions of air). Interpretation is further confounded by FDG within pulmonary blood, which in the healthy lung is substantially larger (typically 15–20%) than other organs, e.g. the brain (typically 5%). Static measures, such as the standard uptake value (SUV), are likely to be heavily influenced by these factors which has led to the exploration of alternative methods that account for these effects [4].

**Fig. 1:**
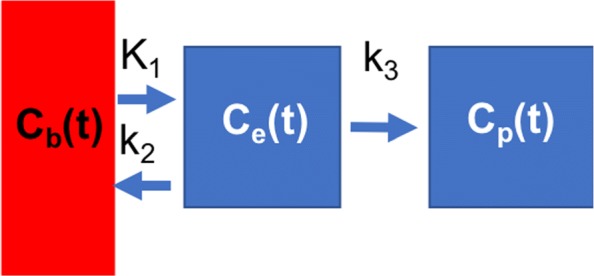
Irreversible two compartmental model describing the kinetics of ^18^F-FDG used to evaluate lung inflammation. In the absence of significant oedema, the concentration of ^18^F-FDG in a ROI in the lung can be described by three compartments [9]: a blood compartment *C*_*b*_(*t*), an extravascular pre-cursor pool *C*_*e*_(*t*) and phosphorylated (’trapped’) ^18^F-FDG compartment *C*_*p*_(*t*). The relationships between the concentration of tracer in a compartment is described by the rate constants (i.e. *K*_1_,*k*_2_,*k*_3_). *ROI* = *Region of Interest*

Kinetic modelling has traditionally been regarded as the gold standard method of quantification in PET studies; well established applications include estimation of cerebral metabolic rate (CMR) and neurological receptor binding (a general framework for kinetic modelling is described in [5]). In the lung, kinetic modelling has recently been used to explicitly account for the effects of air and blood on the rate of ^18^F-FDG uptake [6, 7] which may lead to a better estimation of underlying inflammation. Provided there is not significant oedema, the two compartment irreversible model [8] has been widely adopted to model the kinetics of ^18^F-FDG in pulmonary inflammation [9] (see Fig. 1).

The concentration of ^18^F-FDG measured in a region of interest (ROI) within the lung can be described by:

1 $$ C_{m}(t) = V_{b}C_{b}(t) + (1-V_{a}-V_{b})C_{t}(t, K_{1}, k_{2},k_{3}, V_{b}) + V_{a}C_{a}(t)  $$

where *C*_*m*_(*t*) is the concentration of ^18^F-FDG in the ROI; *C*_*b*_(*t*) and *C*_*t*_(*t*) are the concentrations of ^18^F-FDG in the pulmonary blood vessels and lung cells respectively. *V*_*a*_ and *V*_*b*_ are the fractional volumes of air and blood respectively. *K*_1_,*k*_2_ and *k*_3_ are the microparameters of the model [5]. The concentration of radioactivity in air, *C*_*a*_(*t*), is assumed negligible. *V*_*a*_ can be derived from a CT image as described in [10].

The metabolic rate constant of ^18^F-FDG is then given by

2 $$ K_{{im}} = K_{1}k_{3}/(k_{2}+k_{3})  $$

The microparameters and *V*_*b*_ are estimated by minimising the weighted residual sum of squares (WRSS):

3 $$ {WRSS} = \sum_{i=1}^{N} W_{i} (Y(i) - C_{m}(i))^{2}  $$

where *N* is the number of time frames of the dynamic scan, *i* is the frame number and *W*_*i*_ is the weighting factor for each frame, *C*_*m*_(*i*) is the estimated concentration of ^18^F-FDG fitted from the compartmental model in frame *i* (i.e. Eq. 1) and *Y*(*i*) is the measured concentration from the PET scanner.

An essential requirement of this approach is an accurate blood input function (*C*_*b*_(*t*)) [11]; although arterial blood samples taken during the PET scan are considered the gold standard, obtaining samples from a peripheral vein is less invasive and onerous. Alternatively, a blood TAC can be derived from the dynamic PET images by delineating a ROI within a vessel in the field of view, referred to as an image-derived input function (IDIF). Optimal positioning of the blood vessel ROI has been discussed extensively in application to ^18^F-FDG tumour kinetics [12] and cardiac metabolism [13]; IDIFs derived from several different vessels were found to be comparable to arterial samples. In pulmonary ^18^F-FDG kinetics, several regions have been explored including the ascending and descending pulmonary aorta, left and right ventricles and aorta [14, 15]. The TAC extracted from the blood pool ROI is then modelled as a continuous curve to reduce noise: models based on an initial linear rise followed by a sum of exponentials have been proposed in tumour kinetics [16]. In the brain, extensive effort has been made to improve input function modelling including those based on reference regions [17] and methods using carotid or other blood vessels with one or more manual samples [18–20] with voxel-based approaches also feasible [21]. In the lung, there remains a need for optimisation of input function modelling.

Early studies of kinetic models of cerebral blood flow (CBF) demonstrated the importance of correcting the blood input function for time delay [22] (i.e. the time taken for the tracer to travel between the blood sampling site and the tissue of interest). Methods to estimate the delay between blood sample point and the tissue ROI are largely based on these earlier observations in neurological PET [23, 24]. More recently, inaccuracies in time delay were shown to cause significant deviations in the microparameters of a CMR kinetic model in rodents [25]. This applies equally to pulmonary compartmental models of ^18^F-FDG: incorporating a regional lung time delay has been shown to improve the fit to the experimental data compared to no delay in acute lung injury (ALI) [26]. Further corrections such as accounting for the partial volume effect (PVE) and spill-over in pulmonary ^18^F-FDG scans may be important [15], but the impact of this approach on kinetic parameter estimation in humans has yet to be explored.

The approach taken to model the input function and estimate the time delay are clearly operator dependent with several different feasible methodologies. Further, estimation of the microparameters in Eq. 2 depends on the chosen optimisation algorithm and the applied variance model (i.e. choice of *W*_*i*_) [27]—these too are not yet standarised in pulmonary compartmental models. Experts in quantitative ^18^F-FDG lung imaging have recently highlighted the need to improve the standardisation of the analysis and assess the reproducibility of the outcome parameters (e.g. *K*_*im*_ and *V*_*b*_)[2, 28].

**Fig. 2:**
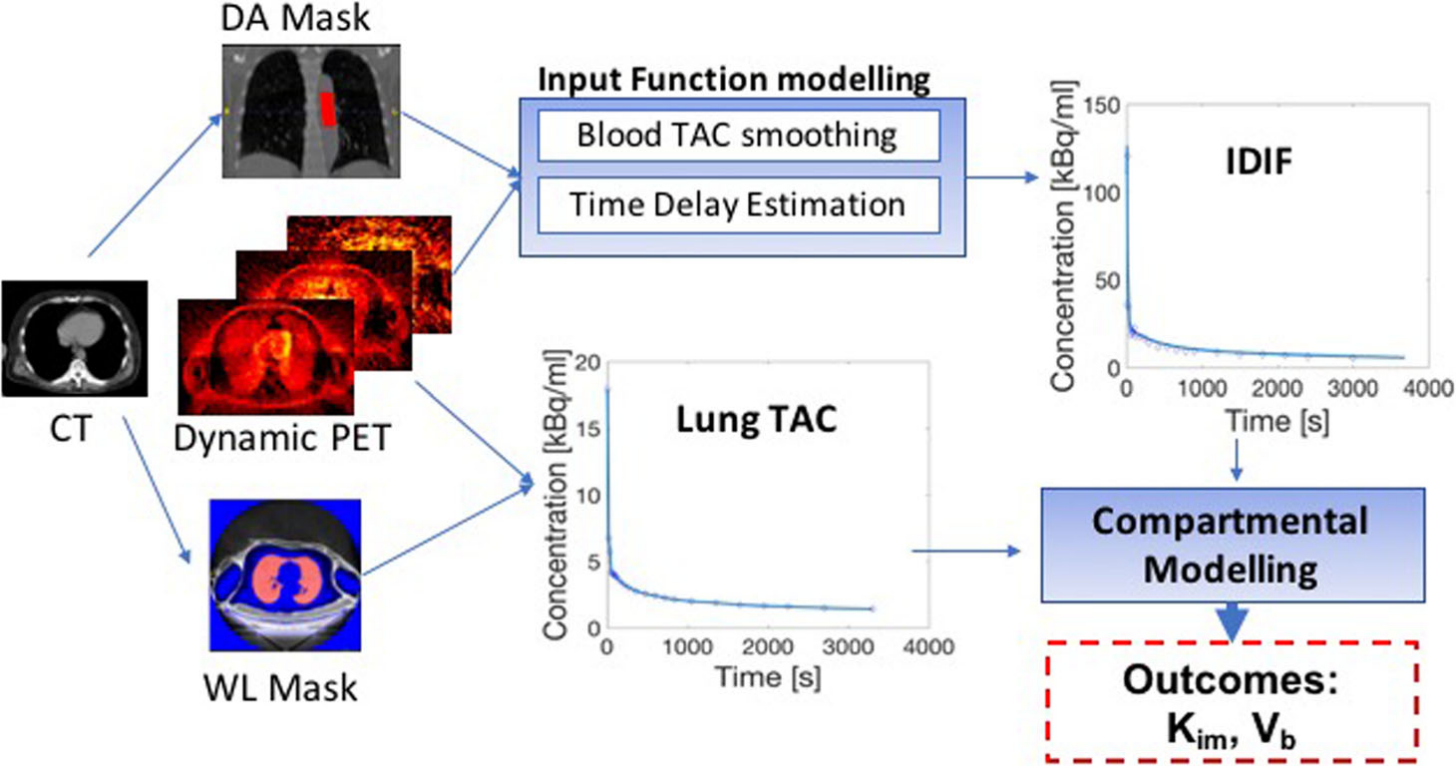
Overview of the main stages of compartmental modelling used in ^18^F-FDG in diffuse lung disease. *DA* = *Descending Aorta*, *WL* = *Whole Lung*, *TAC* = *Time Activity Curve*, *IDIF* = *Image Derived Input Function*

To this end, our aim was to evaluate the reproducibility of pulmonary compartmental models—this has yet to be performed in ^18^F-FDG models of lung inflammation and, to our knowledge, reproducibility of kinetic modelling outcomes in neurological PET also has yet to be disclosed. Reproducibility will be influenced by the number of operator dependent steps required to estimate *K*_*im*_ and *V*_*b*_ (see Fig. 2) and the inherent variability associated with measurements of low signal. We investigate the reproducibility of the analysis with two operators who independently analysed 30 lung scans using different analysis pipelines. During the evaluation, we identified the key parameters in the analyses which could be standarised to help improve reproducibility in pulmonary compartmental modelling of ^18^F-FDG.

## Methods

### Study data

Thirty age and gender matched patients from the EVOLVE study were included in this evaluation: ten patients with COPD (age =66±9; 8M/2F), ten patients with *α*_1_ATD (age =63±8; 7M/3F) and ten healthy never smokers (age =68±8; 9M/1F). The EVOLVE study was a cross-sectional multi-centre study to investigate vascular and pulmonary inflammation (REC 13/EE/0165, UK CRN ID 1513); the primary outcomes of this study are reported in [29]. Patients were clinically diagnosed with COPD stratified with fibrinogen ≤2.8g/L. Healthy volunteers (HV) were recruited if they had no regular smoking history and normal predicted spirometry (further protocol details are available [30]).

### Imaging protocol

A single bolus of approximately 240MBq ^18^F-FDG was injected into the antecubital vein and images were acquired for 60 min under list mode acquisition (further details are available in the Additional file 1). A low dose attenuation correction CT (CTAC) was acquired under free breathing prior to PET scanning. Three subjects (1 COPD, 2 HV) were removed from subsequent analysis after failing initial quality control procedures (see Additional file 1). The blood input function was calculated using an IDIF (as described in the following section); discrete venous blood samples were obtained as a means to calculate a plasma-over-blood (POB) ratio.

### Imaging analyses

For the initial analysis, subject data were analysed by operator A (as described in [7]) using a Molecular Imaging and Kinetic Analysis Toolbox (MIAKAT^TM^ [31]; Version no: 4.2.6) based pipeline—herein referred to as pipeline A. Operator B independently analysed the same dataset using an in-house developed pipeline using MATLAB [32]—herein referred to as pipeline B.

The pipelines shared common procedures for pre-processing: the segmentation of the whole lung and blood vessels were performed using ITK-SNAP (www.itksnap.org [33]), and blood TACs were obtained from an ROI manually delineated within the descending aorta (DA), drawn in the centre of the vessel to minimise the partial volume effect. Our rationale for using the DA is based on previous work which revealed it to be preferable in estimating kinetic rate constants [15] and based on its previous use [7]. The principle differences between the pipelines are outlined in Table 1.

**Table 1:** Comparison of two analysis pipelines used to estimate metabolic rate of ^18^F-FDG to assess pulmonary inflammation

Parameter	Pipeline A	Pipeline B: initial	Pipeline B: final
Blood ROI size	Circular, 5 pixel diameter, aortic arch to variable	Circular, 8 pixel diameter, 25 slices beginning aortic arch	As pipeline A
Lung ROI closing and erosion operation	5 pixel diameter disc	3 pixel diameter disc	As pipeline B initial
Input function model	Exponential basis functions	Tri- or biexponential fits	As pipeline A
Time delay estimation	Inside compartmental model optimisation	Outside compartmental model optimisation	As pipeline A
Time delay fitting	Delays spanning −50 to 50 s using 1-compartmental model fitted for first 5 min—lowest residual sum of squares	Additional parameter within estimation of rate constants	As pipeline A
Optimisation	Local optimum	Global optimum	As pipeline A
Start point of optimisation	*K*_1_ = 0.5, *k*_2_ = 0.2, *k*_3_ = 0.3, *V*_*b*_ = 0.1	Multiple start points generated finds best guess (lowest objective function value)	As pipeline A

The columns “Pipeline B: initial” and “Pipeline B: final” describes the parameters which were used in the initial evaluation and the final settings used following adjustments to pipeline B respectively. The table highlights the key differences between the implementations of the compartmental model. Parameters not included below were identical between the analysis pipelines. *WL* = *whole lung*, *DA* = *descending aorta*, *ROI* = *region of interest*

In pipeline A, subsequent analysis was performed using a MIAKAT-based pipeline: MIAKAT software was modified for lung ^18^F-FDG kinetics by operator A. Importantly, both pipelines used the same compartmental model as the basis to estimate the metabolic rate of ^18^F-FDG (*K*_*im*_) and the fraction blood volume (*V*_*b*_)–the main outcome parameters for this study.

In order to understand the drivers of any differences in the results, operator B investigated the salient parts of the analysis methodology, which led to the differences and adjusted elements of pipeline B (described in the Further investigation section) to improve agreement.

### Statistics

Bland-Altman plots were used to compare the different outcome variables; medians, inter-quartile ranges and correlations were also used. The Bland-Altman coefficient of reproducibility is given by 2× SD; we expect 95% of the difference to be less than this value. The differences in *K*_*im*_ and *V*_*b*_ between the two pipelines were plotted as a histogram to ensure that a normal distribution was observed. To investigate group differences the Hedge’s *g* effect was used, as a further complementary measure we used the unpaired *t* test. Unless otherwise stated, significance is considered when *P*<0.05. The intra-observer repeatability was assessed using the coefficient of variation (COV) by operator B using pipeline B.

## Results

**Fig. 3:**
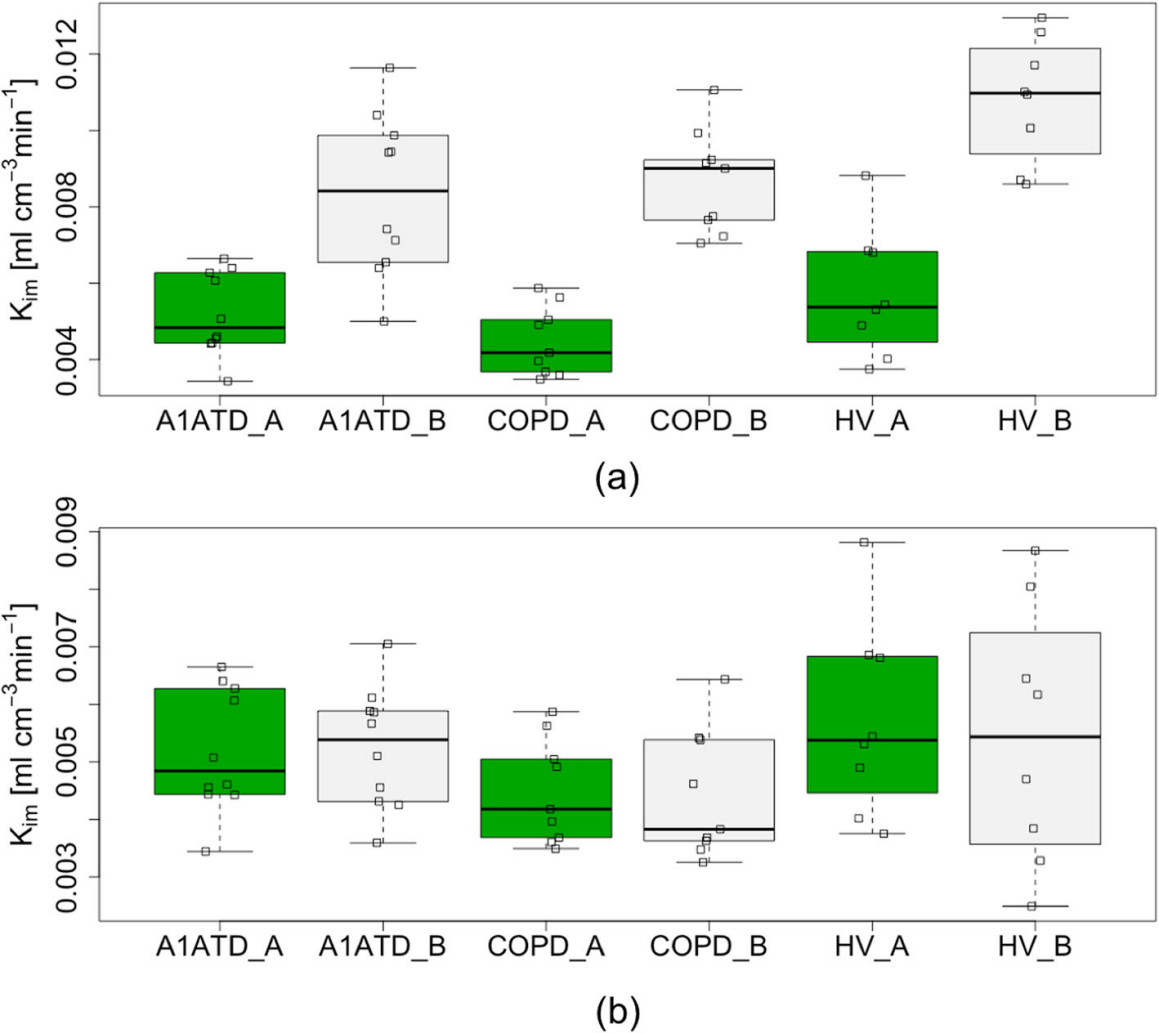
Boxplot of group differences in *K*_*im*_ between the two analysis pipelines. (**a**) Initial comparison between *K*_*im*_ between the two analysis pipelines. (**b**) Comparison between *K*_*im*_ between the two pipelines after all adjustments to Pipeline B (see section Further investigation).*COPD = Chronic Obstructive Pulmonary Disease, A1ATD = α 1-antitrypsin deficiency patients, HV = Healthy Volunteer, _A = Pipeline A result, _B = Pipeline B result*

**Fig. 4:**
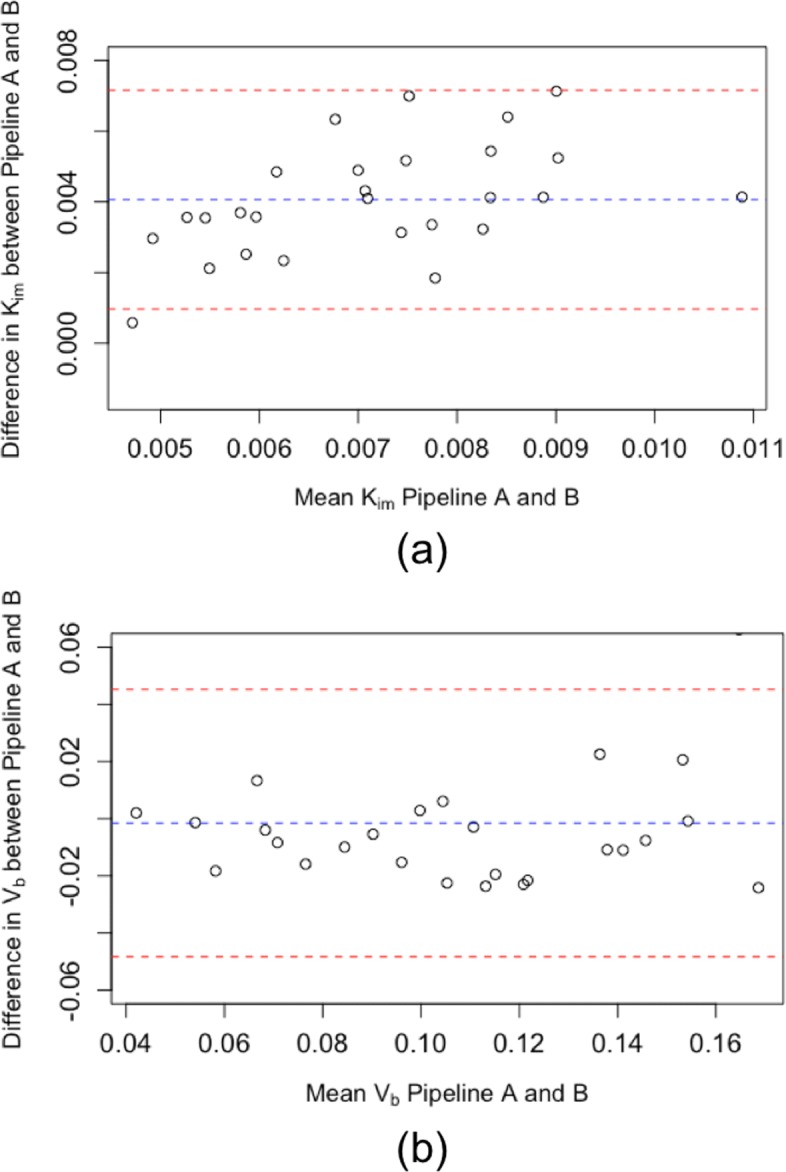
Bland-Altman plots comparing outcome parameters of a pulmonary compartmental model of ^18^F-FDG. (**a**) *K*_*im*_ -the metabolic rate constant of FDG. (**b**) *V*_*b*_ -the fractional blood volume. These are the initial results using two different analysis pipelines. Adjustment of pipeline B led to improved agreement between the pipelines (see Fig. 6))

Initial results are shown in Fig. 3a; boxplots show the differences in *K*_*im*_ between COPD, *α*_1_ATD and controls using the two pipelines. Although a systemic offset was observed between the values obtained between the pipelines, this did not alter the overall group-level conclusions: the Hedge’s *g* factor for the difference between COPD and HV was −0.89 for pipeline A and −0.57 for pipeline B. Further, for pipelines A and B, no significant difference was found between these groups using the two sample *t* test (*p* = 0.088 and *p* = 0.26, respectively). The variance in pipeline B was greater than pipeline A in the *α*_1_ATD group (*IQR*=0.0031 vs 0.0018, respectively). There was poor agreement in *K*_*im*_ on a subject level between the pipelines: Fig. 4a shows the Bland-Altman plot for *K*_*im*_. The mean difference in *K*_*im*_ between the two platforms was 0.0041 ml·c*m*^−3^·mi*n*^−1^ with upper and lower limits of agreement (uloa and lloa) of 0.00097 and 0.0072 ml·c*m*^−3^·mi*n*^−1^ respectively; the correlation coefficient was 0.62. Figure 4a indicates a systematic relationship may exist between the difference and the mean values of *K*_*im*_ estimated using the two pipelines. Following the approach suggested in [34] to reduce systematic bias, we log transformed the data, giving a mean *K*_*im*_ of 1.8, lloa of 1.19 and uloa of 2.73 (after transformation back to the original scale). Although the transformation improved the situation, the agreement between pipelines is still poor. Figure 4b shows the Bland-Altman plot for *V*_*b*_. The mean difference in *V*_*b*_ was −0.0015 (uloa = 0.045, lloa = −0.048); the correlation coefficient was 0.80. The Bland-Altman coefficients of reproducibility were 0.0031 and 0.047 for *K*_*im*_ and *V*_*b*_ respectively. To assess the repeatability of analysis, ten subjects (from COPD group) were analysed five times with pipeline B (operator B): the within subject SD of *K*_*im*_ was 8.28×10^−4^ml·c*m*^−3^·mi*n*^−1^ and the COV was 6.1*%*.

### Further investigation

Subsequently, we sought to understand the drivers of the difference described above; this section describes the steps we undertook. Firstly, visual inspection of the lung tissue TACs from pipeline A and B revealed minimal differences. Further, the mean square error (MSE) between lung TACs from pipeline A and pipeline B was 0.11±0.040; therefore, we concluded that the lung TACs were not responsible for the differences.

Next, we undertook a visual inspection of the blood TACs, this revealed slight differences due to the ROI methodology (e.g. size and location) used by the two operators. To improve agreement, we applied the same DA ROI methodology: in pipeline B, we reduced the area of each ROI and ensured the same begin and end locations in the axial slice as pipeline A. This led to an improvement in the visual comparison of the blood TACs and a modest improvement in *K*_*im*_ (mean difference between was 0.0037 *ml*·*cm*^−3^·*min*^−1^ compared to the original value of 0.0041 ml·c*m*^−3^·mi*n*^−1^). The modelling of the blood TAC was then compared: we found the fitting of the input functions differed chiefly in the early stage of the scan (< 5 min post-injection)—likely due to the inherent noise due to short frame durations. In pipeline B, we altered the input function modelling to match the approach adopted in pipeline A. Firstly, we applied the same fitting function: here, the blood TAC is modelled by basis functions [35] with a varying number of exponentials; the exponential model that best fits the blood TAC was found using a least squares algorithm. With both pipelines using this approach, the Bland-Altman coefficients of reproducibility were modestly improved (0.0023 for *K*_*im*_ and 0.034 for *V*_*b*_).

The time delay estimation is also highly dependent on the initial time frames of the input function; we found an association between the difference in outcome parameters, particularly *V*_*b*_, and the estimated time delay. The mean difference in time delay estimation between pipeline A and B was 3±5.3 s. To improve the agreement between the estimated time delays for each subject, we replicated in pipeline B the method outlined in [7] (used in pipeline A). Namely, a one compartmental model was fitted to the first 5 min of the smoothed blood TAC and lung TAC. Then, a delay of ±50 s in 1s increments was introduced and the delay was estimated by finding the minimum value of the residual sum of squares on the model fit. The mean difference in time delay estimation was improved to 0.76±1 s. This improved the Bland-Altman coefficient markedly to 0.016 for *K*_*im*_ and 0.028 for *V*_*b*_.

**Fig. 5:**
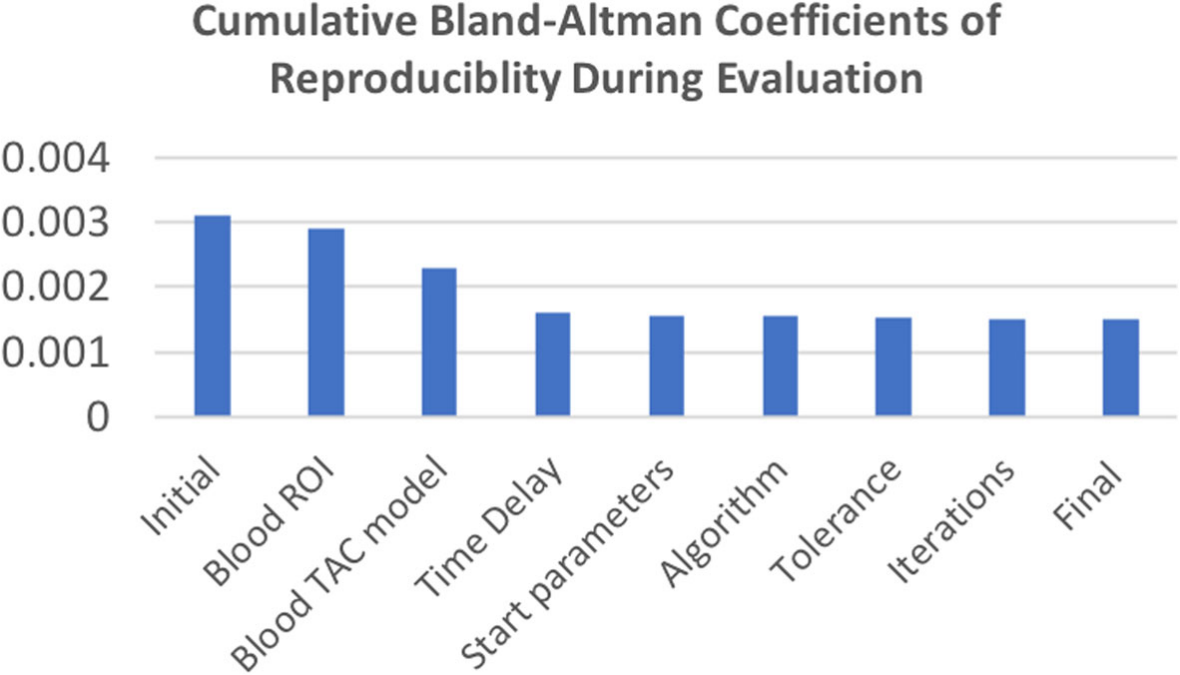
Cumulative Bland-Altman coefficients of reproducibility for *K*_*im*_ during the evaluation of pipeline A and B. Pipeline B was altered at each stage to improve the agreement in *K*_*im*_, each value represents the cumulative effect of all preceding stages. *ROI* = *region of interest*

**Fig. 6:**
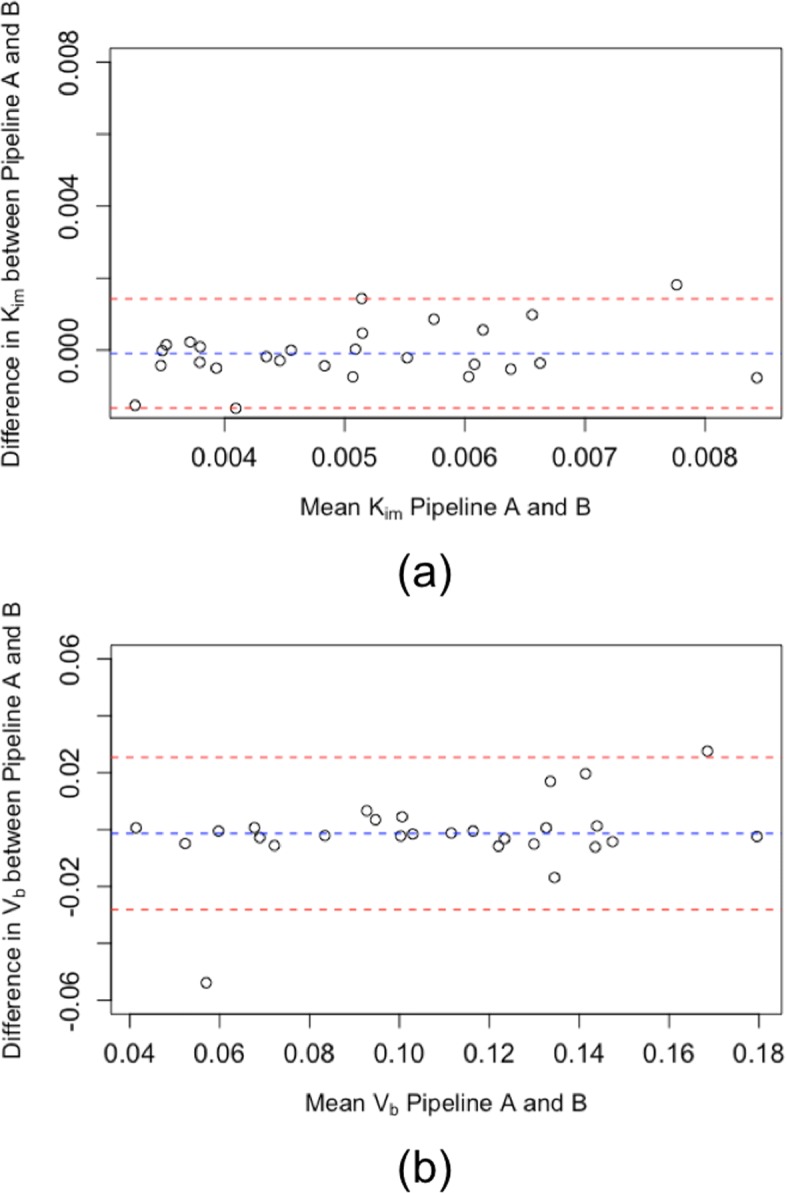
Bland-Altman plots comparing outcome parameters of a pulmonary compartmental model of ^18^F-FDG using two independent analysis pipelines following adjustment of pipeline B. (**a**) *K*_*im*_ -the metabolic rate constant of FDG. (**b**) *V*_*b*_ -the fractional blood volume. This should be compared to the initial results in Fig 4

Various factors were adjusted in the optimisation algorithm including the function, number of iterations, tolerance and initial parameter. But these were found to have less influence on outcome parameters. Figure 5 summarises the incremental improvement in agreement during each stage of the evaluation as we altered pipeline B. Following all adjustments to pipeline B, the Bland-Altman plots are shown in Fig. 6; the mean differences were *K*_*im*_=9.0×10^−4^ ml·c*m*^−3^·mi*n*^−1^ and *V*_*b*_=−0.0014 with coefficients of reproducibility of 0.0015 and 0.027. The correlation was also improved: for *K*_*im*_,*r*=0.86 and for *V*_*b*_,*r*=0.94. Figure 3b demonstrates the improvement in agreement on a group level. *K*_*im*_ calculated using the adjusted pipeline B had larger variability than pipeline A in the healthy control group (*IQR*=0.0031 vs 0.0022, respectively); we did not observe any notable change in the other groups (see Fig. 3).

## Discussion

We investigated the reproducibility of pulmonary compartmental modelling of inflammation using two independent analysis pipelines; *K*_*im*_ and *V*_*b*_ were the main outcome parameters of this study, *K*_*im*_ was interpreted as a surrogate for lung inflammation. Two independent operators used reconstructed dynamic PET and CT scans from the same 30 subjects. Initial results showed that the subject-level agreement in *K*_*im*_ and *V*_*b*_ was poor; despite the application of the same kinetic model (i.e. Eqs. 1 and 2). Interestingly, this did not change the overall interpretation of the group findings; the effect size between groups was comparable using either pipeline. Further, it did not change the outcome of statistical hypothesis testing between groups. Reproducibility of analysis is an important pre-requisite for an imaging biomarker; the results of this evaluation demonstrate the need for standardisation when applying compartmental modelling to assess lung inflammation.

The excellent repeatability of pipeline B (COV =6.1*%*) indicates that the poor agreement between the pipelines is likely to be due to the implementation of the compartmental model. To investigate the salient stages responsible for the differences, we used an incremental approach: beginning with the steps involved in generating the TACs and all subsequent stages to estimate *K*_*im*_ and *V*_*b*_. Visual comparison of the blood input functions revealed that the largest differences seemed to be in the stages of extracting and modelling the blood input functions. We determined that the salient factors were the blood ROI methodology, input function modelling and time delay estimation. ROI definition led to differences in estimates of concentration due to presence of the PVE - neither pipeline applied corrections for PVE; it has been suggested this could also help reduce bias in the kinetic parameters estimation [9], but this has yet to be confirmed in human studies. Although adjusting the ROI size did improve the agreement between the pipelines modestly, agreement was improved substantially by the subsequent modelling of the input function.

Modelling the input function provides a means to minimise the noise and spillover problems in PET measurements [36, 37]. Various models exist, common examples include the Feng model [36] and tri-exponential equations, which use regression to estimate the best fit to the blood TAC. In these data, we found the fitting was particularly sensitive to the first few time frames—corresponding to the images with the largest noise content. There are several proposed methods to calculate the time delay between the blood input function and the tissue of interest. We found large differences in estimates of time delay between pipelines A and B, given its sensitivity to the first few minutes of data collection this is clearly exacerbated if the input function models produce different fits in this region.

By adjusting the blood ROI definitions, the input function fitting method and the time delay calculation in pipeline B, we were able to demonstrate better agreement between the two pipelines (see Figs. 3b and 6). Such parameters are often not reported in the “Methods” section of existing literature; these findings highlight the need for reporting details of analysis methodology to facilitate the reproducibility of outcomes in compartmental models of the lung. Adjustment of pipeline B led to higher variability in *K*_*im*_ in the HV group. It seems likely that this is due to the higher noise content in HV scans, which may have led to more extreme differences in fitting of the IDIF. Future work is needed to determine the importance of this variance in pulmonary compartmental models.

The POB ratio was calculated for each subject using discrete venous blood samples; this allows the conversion of a blood input function to a plasma input function. Although the same factors were used for both pipelines, we recognise that this may cause bias in the outcome variables. We found the POB ratio to be very close to one (1.05±0.03); this agrees with previous findings that ^18^F-FDG equilibrates between erythrocytes and plasma nearly instantaneously [38]. Further work should determine whether POB corrections are necessary in quantitative ^18^F-FDG lung studies. Previous work has demonstrated that the weighting factors chosen to describe the variance of dynamic PET data affects the outcomes of compartmental modelling [27]; here, the weighting factors were identical in both pipelines and we did not explore the impact of different weighting schemes but acknowledge this could be another key contributor which may improve reproducibility. Respiratory motion leads to both density variations in the lung and inaccuracies in the attenuation correction applied to the PET image; both of these influence quantitative PET and improvements have been suggested by use of an averaged CT scan when available [4]. Since we acquired the CT scans during free breathing, these inaccuracies may have an influence on our quantitative PET data. This study investigated the reproducibility of pulmonary compartmental models of dynamic ^18^F-FDG PET/CT; since both operators used the same reconstructed datasets, this does not include within subject biological variability or technical factors (e.g. scanner settings and reconstruction algorithms), which would allow the overall reproducibility of pulmonary ^18^F-FDG scans to be evaluated. Nevertheless, with each operator using identical scans, we avoid the systematic variability in ^18^F-FDG uptake introduced by differences between hardware, reconstruction and acquisition protocols [39]. We were able to assess the intra-observer agreement for pipeline B (operator B); we found this to have excellent repeatability. We could not explore the inter-observer effect as results from pipeline A, undertaken by operator A, were retrospectively acquired. Yet, we were able determine which stages of compartmental modelling were responsible for the low concordance observed between the pipelines.

Currently, there is no standard method of assessing pulmonary inflammation using compartmental models of ^18^F-FDG and each centre may undertake such analyses using their own bespoke approach. Our findings suggest that despite seemingly similar methodology, individual subject results are sensitive to several factors in the analysis; therefore, care is needed when reporting the exact methods used. In our lung data, we identified that the blood ROI methodology, IDIF modelling and time delay estimation were important drivers of reproducibility. In light of these findings, we suggest that any forthcoming recommendations for reporting methodology incorporate these key features.

## Conclusions

Reproducibility of pulmonary compartmental modelling of ^18^F-FDG PET-CT was evaluated in 30 subjects consisting of three groups: COPD patients, *α*_1_ATD patients and healthy never smokers. Two operators analysed the imaging data using independent software to estimate the metabolic rate constant of ^18^F-FDG and fractional blood volume. Initial comparisons showed good agreement in the overall conclusions at group level; however, subject-level agreement was poor. We identified salient factors in the analysis, which improved the agreement between the two analysis pipelines: blood ROI methodology, input function modelling and time delay estimation. In a field where in-house analyses are commonplace, these findings highlight the need for standardisation in reporting of methods, which will help to improve the reproducibility.

## Supplementary information

**Additional file 1** Online supplement.

## Data Availability

The datasets used for and generated during the analysis are available on reasonable request from the corresponding author upon completion of a material transfer agreement. MIAKAT^TM^ [31] is freely available but requires modification for lung analysis.
