# Supplementary material for: Reproducibility of compartmental modelling of 18F-FDG PET/CT to evaluate lung inflammation
Source: EJNMMI Phys. 2019 Dec 16;6:26. doi: 10.1186/s40658-019-0265-8 (PMC6915187; doi:10.1186/s40658-019-0265-8)
Supplement: Supplementary file 1 — Additional file 1 Online supplement. [file 40658_2019_265_MOESM1_ESM.docx]

Supplementary Data

**Methods**

**Acquisition**

All subjects were scanned at two sites: Cambridge University Hospitals NHS Foundation Trust using a GE690 PET/CT scanner or at Invicro, Hammersmith using a Siemens Biograph TruePoint 6 PET-CT scanner. Participants were told to fast for 6 hours prior to the scan, a blood glucose test was performed and if > 11mmol participants were rescheduled. Prior to the PET scan participants underwent an CT Attenuation Correction (CT-AC) scan; this was acquired under normal breathing. ^18^F-FDG was administered intravenously in the antecubital vein; the target administered activity was 240MBq. Venous blood samples were drawn from the contralateral arm at 12 time points for the POB correction (at 1, 2, 3, 4, 5, 7, 10 , 15, 20, 30, 45 and 60 minutes). PET data was acquired under list mode and binned into 23 frames (8x15s, 3x60s, 5x120s, 5x300s, 2x600s); histograms were reconstructed at either 2mm nominal slice thickness using DIFT (Siemens) or 3.27mm nominal slice thickness using 3D Fore FBP (GE). Corrections for attenuation, deadtime, decay and scatter were incorporated. The DICOM files were converted into a single 4D Nifti file for analysis.

**Analysis**

Semi-automated segmentation of the whole lung (WL) from the CT-AC images was achieved using either ITK-SNAP (in Pipeline B) or Analyze 11.0 (AnalyzeDirect, Inc., Overland Park, KS - Pipeline A). Intensity thresholds of -1022 and -380 were used. The WL mask was then checked for obvious artifact and manually modified. On a slice-by-slice manner, starting at the apex, the operator followed the trachea to the interface between primary bronchi and lung parenchyma: any voxel corresponding to the airways initially included in the WL mask was then excluded.

PET and CT images were resliced to yield isotropic voxel size (2 x 2 x 2 mm^3^ for PET and 1.37 x 1.37 x 1.37 mm^3^ for CT-AC ). CT-AC and WL mask were then downsampled to match the PET voxel size. Excessive movement was determined by visual assessment of an experienced reader. A closing operation was performed on WL mask using either a five voxel (pipeline A) or 3 voxel (pipeline B) diameter disks. Subsequently, an erosion operation was performed on the mask using a five voxel or 3 voxel diameter disks to remove artifact near the borders of the lung. Further, a manual erosion was performed to remove any remaining artifact at diaphragm level based on a visual assessment of the PET uptake.
